# Selective Conservatism in the Management of Spinal Stab Wounds Remains Applicable—A Critical Review of 169 Patients Managed at a Major Trauma Center in South Africa

**DOI:** 10.1002/wjs.12685

**Published:** 2025-06-26

**Authors:** Reuben He, Victor Kong, Cynthia Cheung, Jonathan Ko, Daniel Lee, Joshua Ahn, Shuba Kosna, William Yeung, Hong Lee, Howard Wain, Wanda Bekker, Damian Clarke

**Affiliations:** ^1^ Department of Surgery Faculty of Medical and Health Sciences The University of Auckland Auckland New Zealand; ^2^ Department of Surgery University of the Witwatersrand Johannesburg South Africa; ^3^ Department of Surgery Wellington Regional Hospital Newtown New Zealand; ^4^ Department of Surgery Royal Adelaide Hospital Adelaide Australia; ^5^ Department of Surgery University of KwaZulu‐Natal Durban South Africa

**Keywords:** orthopedic surgery, spine, trauma surgery

## Abstract

**Background:**

Spinal stab wounds (SW) are relatively uncommon and can be both morbid and fatal. The exact role of surgery remains somewhat unclear. This study reviews our institutional experience of spinal SW management and examines the clinical outcome of these patients in a developing world setting.

**Methods:**

A retrospective study was conducted over a 10‐year study period from December 2012–December 2022 at a major trauma center in South Africa. All patients who sustained spinal SW were included.

**Results:**

One hundred sixty‐nine patients with spinal SW were included (male: 87%, mean age: 28 years, median Injury Severity Score [ISS]: 9). AIS classifications: E (51%), C (18%), A (14%), D (12%), B (4%). Nearly all patients (95%) underwent CT scan and 81% had an MRI. 72% had bony injury and 60% had spinal cord injury. Five percent of all patients underwent surgical intervention. Overall, 5% of patients required intensive care unit (ICU) admission and 9% had one or more complications. Common complications were hospital acquired pneumonia and pressure sores. The overall in hospital mortality rate was 1% (2/169). Of the 167 patients who survived to hospital discharge, 88% were discharged to spinal rehabilitation centers and the remaining patients were discharged home.

**Conclusions:**

Our study supports a conservative management in select SW patients, especially in the absence of progressive neurological deficits or spinal instability. Non‐operative management aligns well with resource‐constrained public healthcare facilities in our environment. Further research should aim to develop context‐specific guidelines to refine surgical decision‐making and improve outcomes across varied healthcare settings.

## Graphical abstract

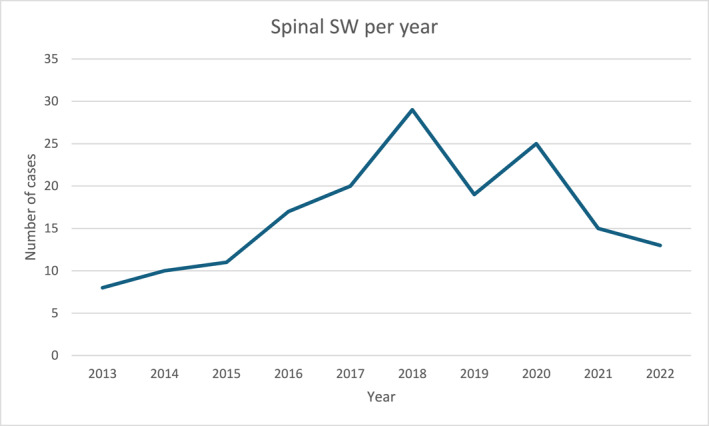

## 1 Introduction

South Africa has the dubious distinction of having one of the highest rates of interpersonal trauma in the world. Knife‐related assault is extremely common in South Africa and can be both morbid and fatal. In response to this pandemic of knife related trauma, a voluminous body of literature discussing the management of penetrating injuries has emanated from the country over the last 50 years [1, 2]. Despite this commendable output, there are injuries which have tended to be underreported or overlooked. One such area is the issue of knife related spinal cord injury. With the country's high incidence of knife related trauma, spinal SWs contribute disproportionately to the etiology of spinal cord injury in South Africa in comparison to other high‐income countries [3, 4].

Knife related spinal injuries are a clinically significant variant of spinal trauma. Unlike in blunt trauma, a spinal SW typically produces a localized trauma which directly injures the spinal cord, nerve roots, dura mater, or vasculature of the spinal cord [5, 6]. This can occur without associated vertebral fracture or gross instability [5]. These injuries present in a heterogenous fashion, ranging from asymptomatic patients to those with complete neurological deficits [7]. As such, spinal SWs pose diagnostic and therapeutic challenges due to their variable presentation and potential for rapid neurological deterioration. A spinal SW is a low‐velocity form of penetrating trauma, inflicted with a knife or sharp object. The extent of neurological injury depends on several factors, including the depth and trajectory of the blade, the spinal level involved, and the presence of associated complications such as a hematoma, spinal cord laceration, vascular injury, or cerebrospinal fluid (CSF) leak [5, 8, 9]. The spinal cord may be injured directly by laceration or indirectly through vascular compromise, dural penetration, or secondary edema. Neurological deficits can range from complete spinal cord injury to subtle radiculopathies or may be entirely absent in cases where the cord or roots are spared [7]. The absence of a bone injury does not preclude severe neurological compromise [10]. Retained foreign bodies, such as knife fragments, is an additional concern that can complicate management and the clinical course.

Management of a spinal SW is mostly non‐operative and expectant [11]. Non‐surgical management such as observation, antibiotic prophylaxis, and immobilization is appropriate in neurologically intact patients or those with a neurological deficit which is stable and in the absence of a retained foreign body, CSF leak, or progressive neurological deterioration [12]. Surgical intervention may be indicated where there is a progressive neurological deficit, evidence of a compressive lesion, persistent CSF leak, or penetrating injury with signs of infection or abscess formation [12, 13]. Despite growing literature on the topic, no universal consensus exists, and management is often guided by institutional protocols and clinician experience [5, 14, 15]. The aim of this study was to evaluate the incidence and clinical characteristics of spinal cord injuries resulting from SWs managed at a major trauma center in South Africa, and to analyze clinical outcomes of patients with this injury.

## 2 Materials and Methods

### 2.1 Clinical Setting

The Pietermaritzburg Metropolitan Trauma Service (PMTS) is based at Gray's Hospital based in the city of Pietermaritzburg, South Africa. The PMTS provides definitive trauma care to the city of Pietermaritzburg and the surrounding catchment area with a total population of over three million people. Annual admissions exceed 5000, over 50% of which are related to penetrating injuries. The Hybrid Electronic Medical Registry (HEMR) is our regional electronic trauma database and captures all trauma admission at our institution.

### 2.2 Management Protocol

All patients are initially assessed in the trauma bay in accordance with the *Advanced Trauma Life* *Support* (ATLS) protocol. All penetrating spinal injury trauma patients are reviewed and evaluated according to the American Spinal Injury Association (ASIA) Impairment Scale (AIS) to classify the severity of spinal cord injuries. All patients who sustain a SW are assessed clinically and the extent of injury is evaluated with a combination of clinical examination and plain radiography. Computed tomography (CT) imaging helps assess for bone fractures, retention of foreign objects, and associated organ injuries. If retention of foreign objects is excluded, MRI is utilized for further characterization of the extent of spinal cord injury and surrounding soft tissue damage. MRI is also used for patients with persisting clinical concern for spinal cord injury (e.g., focal neurologic deficit) despite previous normal examinations and/or imaging. Patients who have a spinal injury identified by clinical exam and imaging are referred to spinal and/or neurosurgery for appropriate management.

### 2.3 The Study

Longitudinal 10‐year data (December 2012–December 2022) were extracted from the HEMR trauma database which included all patients with spinal SWs. Ethics approval for the maintenance of our registry and for this study was formally approved by the Biomedical Research Ethics Committee of the University of Kwa Zulu Natal (BCA 221/13).

### 2.4 Statistical Analysis

Descriptive analysis was completed to summarize the raw data. Statistical analysis was performed using SPSS (Version 29, IBM Corp, Armonk, NY, USA).

## 3 Results

### 3.1 Overview

**FIGURE 1:**
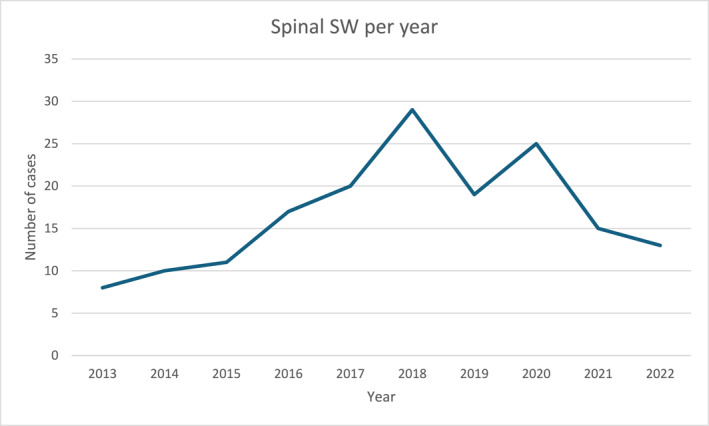
Trend in number of spinal SW per year over study period. SW, stab wound.

During the 10‐year study period, 169 patients sustained a SW to the spine and were included in the study. This represented approximately 4% of all SW over the study period (*n* = 4589). Eighty‐seven percent were male (147/169) and the median age was 28 (interquartile range (IQR) 23–34) years. The median Injury Severity Score (ISS) was 9 (IQR 9–16). Table 1 summarizes the patient characteristics. The most common AIS classifications in descending order were E (51%), C (18%), A (14%), D (12%), and B (4%). Nearly all patients underwent a CT scan (95%) and 81% had an MRI. The trend in the number of spinal SW cases each year steadily increased from 2013 to 2018, reaching a peak of 29 cases in that year. After 2018, this rate has waxed and waned to a 5‐year low of 13 cases in 2022. Figure 1 illustrates the trends for each full calendar year of data.

**TABLE 1:** Characteristics of SW penetrating spine trauma patients.

Variable	Total (*n* = 169)
Median age (IQR)	28 (23–34)
Male	147 (87%)
Median shock index (IQR)	0.7 (0.6–0.8)
Median ISS (IQR)	9 (9–16)
Median GCS on admission (IQR)	15 (15–15)
Median RTS (IQR)	8 (8–8)
ASIA impairment scale	
Complete (A)	24 (14%)
Sensory incomplete (B)	6 (4%)
Motor incomplete (C)	31 (18%)
Motor incomplete (D)	21 (12%)
Normal (E)	87 (51%)
Imaging	
CT	161 (95%)
MRI	137 (81%)

Abbreviations: ASIA, American Spinal Injury Association; CT, computed tomography; GCS, Glasgow coma scale; IQR, interquartile range; ISS, injury severity score; MRI, magnetic resonance imaging; RTS, Revised Trauma Score; SW, stab wound.

### 3.2 Injury Spectrum

The spectrum of injury is summarized in Table 2. Overall, 72% of patients suffered an injury to the bony structures of the spine (e.g., fracture, dislocation), with the most common region affected being the thoracic spine (49% of all bony spine injuries). Sixty percent of patients had a spinal cord injury, with the most affected region being the thoracic spinal cord (60% of all spinal cord injuries). All but three patients had at least one concurrent body injury, with high rates of concurrent injury in the thorax (56%), head/neck (53%), and abdomen (18%) regions.

**TABLE 2:** Spectrum of injury in SW penetrating spine trauma patients.

	Penetrating spine trauma
Variable	Total (*n* = 169)
Bony spine injury	121 (72%)
Cervical	50 (30%)
Thoracic	59 (35%)
Lumbar	18 (11%)
Spinal cord injury	101 (60%)
Cervical	34 (20%)
Thoracic	61 (27%)
Lumbar	8 (7%)
Sacral	0 (0%)
Concurrent body injury	166 (98%)
Head/neck	89 (53%)
Face	16 (9%)
Thorax	109 (64%)
Abdomen	31 (18%)
Pelvis	6 (4%)
Limb	26 (15%)

*Note:* one patient may suffer concurrent injuries to more than one body region.

Abbreviation: SW, stab wound.

### 3.3 Management

**FIGURE 2:**
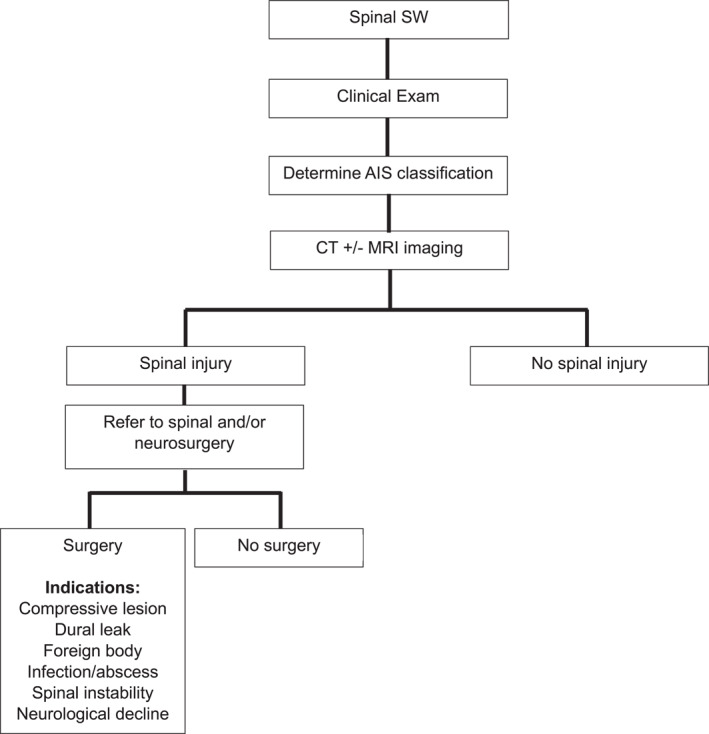
Management algorithm for spinal SW patients. AIS, American Spinal Injury Association Impairment Scale; CT, computed tomography; MRI, magnetic resonance imaging; SW, stab wound.

Of the 169 patients with a spinal SWs, 5% (8/169) underwent surgical intervention. Seven patients underwent laminectomy/decompression/foreign body removal and one patient required fusion/fixation. Of the patients who underwent spinal surgery, three were AIS E (two laminectomy and removal of foreign body, one fixation), two were AIS D (both laminectomy and removal of foreign body), two were AIS C (both laminectomy and dural repair), and there was a single patient with AIS A classification (laminectomy and duroplasty). No patients with AIS B received surgical intervention. Overall, six of the eight patients who underwent spinal surgery had a spinal cord injury. This represented 6% of all patients who had a spinal cord injury (*n* = 101). In total, 15% of patients underwent surgery for concurrent injuries to other body cavities. The most common procedure was a laparotomy in 46% of these patients. The management of SW penetrating spine trauma patients is summarized in Table 3. Based on our results, a suggested management algorithm for patients suffering from spinal SW is demonstrated in Figure 2.

**TABLE 3:** Management of SW penetrating spine trauma patients.

Variable	Total (*n* = 169)
Spinal injury	
Conservative	161 (95%)
Surgical	8 (5%)
Laminectomy/decompression/ROFB	7 (4%)
Fusion/fixation	1 (< 1%)
Concurrent body injury	
Surgical	26 (15%)
Laparotomy	12 (7%)
Thoracotomy/thoracoscopy	3 (2%)
Vascular repair	5 (3%)
Maxfax	1 (< 1%)
ORIF	0 (0%)
Wound closure	3 (2%)
Neurosurgery	2 (1%)
Nerve/tendon repair	2 (1%)
Arthrotomy/joint washout	0 (0%)
Ophthalmology (eye evisceration)	0 (0%)

*Note:* one patient may have more than one surgical procedure done.

Abbreviations: Maxfax, oral and maxillofacial surgery; ORIF, open reduction and internal fixation; ROFB, removal of foreign body; SW, stab wound.

### 3.4 Clinical Outcomes

Overall, 8 (5%) patients required intensive care unit (ICU) admission, with 1 patient (< 1%) requiring ventilatory support. Overall, 15 (9%) patients had complications. The most common complications were hospital acquired pneumonia and pressure sores. The overall in hospital mortality rate was 1% (2/169). For the 167 patients who survived to hospital discharge, 88% were discharged to spinal rehabilitation centers and the remaining patients were discharged home. The outcomes of spinal SW patients is summarized in Table 4.

**TABLE 4:** Outcomes of SW penetrating spine trauma patients.

Variable	Total (*n* = 169)
Complications from management	
Respiratory	4 (2%)
Renal	1 (< 1%)
Abdominal	2 (1%)
Neurological	1 (< 1%)
Wound	2 (1%)
Other	6 (4%)
Intensive care	
ICU admission	8 (5%)
Median ICU stay in days (IQR)	6 (3–13)
Requiring ventilation	1 (< 1%)
Mortality	2 (1%)
Discharge plan	
Rehabilitation	148 (88%)
Home	19 (11%)

Abbreviations: ICU, intensive care unit; SW, stab wound.

## 4 Discussion

Spinal SW remain relatively uncommon and historically, series from the 1960s emanating from South Africa advocated a selective conservative approach to its management [16]. In one of the largest series of spinal SW published by Lipschitz and Block from Baragwanath Hospital in Johannesburg, a significant number associated non spinal injuries were noted [17]. This was consistent with the findings of our studies. The management of spinal SW tends to be non‐operative and there is a low reported rate of surgical intervention globally [5, 14, 15, 18]. This trend is particularly pronounced in our study, where most cases of spinal SW did not undergo surgery. In the select cases that required surgery, patients generally had retained foreign body on imaging posing risk of neurological compromise (*n* = 4), dural leak (*n* = 2), or clinical deterioration (*n* = 2). These were all indications for surgical intervention. Several factors contribute to the low rate of operative management. The majority of spinal SWs do not result in spinal instability or mechanical disruption of bony elements, which are the major reason for surgical stabilization [19]. Spinal SW injuries follow a direct trajectory through the soft tissues. Frequently the spinal cord or cauda equina is either narrowly missed or only partially lacerated [18]. This is especially the case if there is no progression or deterioration in the neurological deficit [20]. Modern imaging modalities, particularly CT and MRI, allow for detailed assessment of spinal cord integrity, presence of foreign bodies, hematoma, or dural injury [21, 22, 23]. Where imaging confirms the absence of compressive lesions, surgical exploration is unnecessary.

In many South African public hospitals, access to imaging, in particular MRI, is limited. This situation has paradoxically encouraged more conservative management protocols and the clinical data suggests that this conservative approach produces results which are comparable if not superior to a more aggressive approach [24, 25]. Surgical exploration of penetrating spinal injuries carries inherent risks, including infection, iatrogenic neurological damage, and prolonged hospitalization. This conservative approach aligns with management strategies in high‐resource settings [5, 18]. However, in high‐resource settings, there is likely to be more timely access to advanced quality imaging, long‐term rehabilitation facilities, as well as surgical specialists and operation theaters for selected operative cases.

Spinal SW injuries are distinct from high‐velocity ballistic injuries in that there is no penumbra of tissue damage and ischemia, and the prognosis in neurologically intact patients is generally favorable [5, 20, 26]. Even in cases of incomplete neurological deficits, many patients improve with supportive care, physical rehabilitation, and close monitoring [27]. In this context, our study's observed mortality rate of 1% in spinal SW cases is notably low compared to traumatic spinal cord injury rates from other developing countries, which varied widely from as low as 1.4% to as high as 20% [28, 29]. The low mortality in our study cohort suggests that, when appropriately selected, conservative management does not compromise patient survival.

However, it should be noted that a limitation in our study was the absence of post‐discharge follow‐up, especially regarding neurologic or functional recovery. This may affect long‐term outcomes data for our patient group who survived to discharge.

There is an ongoing debate in the literature about whether early surgical intervention may improve outcomes in select cases, particularly those with incomplete spinal cord injuries or radiologically confirmed compressive pathology [5, 14, 30, 31]. In South Africa, where high patient volumes and systemic constraints often influence care pathways, there is a need for future multicenter studies to refine operative indications and optimize patient outcomes. Our study findings can inform context‐specific treatment protocols and training priorities for trauma providers by streamlining the management of spinal SW patients in low‐resource settings (Figure 2).

## 5 Conclusion

Our study supports a conservative management in select spinal SW patients, especially in the absence of progressive neurological deficits or spinal instability. This approach also helps to reduce surgical workload and associated risks, making it a pragmatic and sustainable model for trauma care. Clear criteria for surgical referral remain essential, particularly in cases of neurological deterioration or radiological evidence of compression. Further research should aim to develop context‐specific guidelines to refine decision‐making and improve outcomes across varied healthcare settings.

## Author Contributions

**Reuben He:** investigation, writing – original draft, writing – review and editing, methodology, formal analysis, data curation. **Victor Kong:** supervision, writing – original draft, writing – review and editing, conceptualization, project administration, resources. **Cynthia Cheung:** writing – review and editing. **Jonathan Ko:** writing – review and editing. **Daniel Lee:** writing – review and editing. **Joshua Ahn:** writing – review and editing. **Shuba Kosna:** writing – review and editing. **William Yeung:** writing – review and editing. **Hong Lee:** writing – review and editing. **Howard Wain:** writing – review and editing. **Wanda Bekker:** writing – review and editing. **Damian Clarke:** writing – review and editing, supervision.

## Conflicts of Interest

The authors declare no conflicts of interest.

## Data Availability

Data sharing is not applicable to this article as no new data were created or analyzed in this study.
